# Insights of Host Physiological Parameters and Gut Microbiome of Indian Type 2 Diabetic Patients Visualized *via* Metagenomics and Machine Learning Approaches

**DOI:** 10.3389/fmicb.2022.914124

**Published:** 2022-07-18

**Authors:** Debjit De, Tilak Nayak, Subhankar Chowdhury, Paltu Kumar Dhal

**Affiliations:** ^1^Department of Life Science and Biotechnology, Jadavpur University, Kolkata, India; ^2^Department of Endocrinology, Institute of Post Graduate Medical Education and Research (IPGMER) and SSKM Hospital, Kolkata, India

**Keywords:** type 2 diabetes, gut microbiota, machine learning, feature selection, microbial communities

## Abstract

Type 2 diabetes (T2D) is a serious public health issue and may also contribute to modification in the structure of the intestinal microbiota, implying a link between T2D and microbial inhabitants in the digestive tract. This work aimed to develop efficient models for identifying essential physiological markers for improved T2D classification using machine learning algorithms. Using amplicon metagenomic approaches, an effort has also been made to understand the alterations in core gut microbial members in Indian T2D patients with respect to their control normal glucose tolerance (NGT). Our data indicate the level of fasting blood glucose (FBG) and glycated hemoglobin (HbA1c) were the most useful physiological indicators while random forest and support vector machine with RBF Kernel were effective predictions models for identifications of T2D. The dominating gut microbial members *Allopreotella, Rikenellaceae RC9 gut group, Haemophilus, Ruminococcus torques group*, etc. in Indian T2D patients showed a strong association with both FBG and HbA1c. These members have been reported to have a crucial role in gut barrier breakdown, blood glucose, and lipopolysaccharide level escalation, or as biomarkers. While the dominant NGT microbiota (*Akkermansia, Ligilactobacillus, Enterobacter*, etc.) in the colon has been shown to influence inflammatory immune responses by acting as an anti-inflammatory agent and maintaining the gut barrier. The topology study of co-occurrence network analysis indicates that changes in network complexity in T2D lead to variations in the different gut microbial members compared to NGT. These studies provide a better understanding of the gut microbial diversity in Indian T2D patients and show the way for the development of valuable diagnostics strategies to improve the prediction and modulation of the T2D along with already established methods.

## Introduction

Type 2 diabetes (T2D) is a metabolic disorder that affects people all over the world and is caused by both inherited and environmental factors, such as physical inactivity, sedentary lifestyles, cigarette smoking, and excessive alcohol use because these factors create stress on a pancreatic β-cells resulting in decreased insulin sensitivity and production. Due to the β-cell dysfunction, both normal blood glucose level and insulin sensitivity are gradually hampered, resulting in pathophysiological changes and the development of several complications in patients (McIntyre et al., 2019). According to International Diabetes Federation (IDF) report, a total of 415 million people have diabetes globally (as of 2015) and this may increase to 642 million by 2040 because of T2D (Zhang et al., 2013, 2021a; Cho et al., 2018). Several mathematical and statistical models were established using human physiological parameters to predict the disease and/or risk of the disease, machine learning (ML) is one of them. Machine learning is a useful statistical method to analyze high-dimensional and multimodal biomedical data and disease diagnostics (Yu et al., 2020). Several studies endorsed the discrimination between T2D and normal person normal glucose tolerance (NGT) using different ML models based on patients' physiological conditions (Zhang et al., 2021b). However, most of those studied models made their observations based on the limited number of samples from a single geographical location. Additionally, none of them attempted to identify important physiological parameters out of their prediction model that significantly differentiates T2D disease from NGT. While best prediction model with high accuracy essentially needed a large sample size with variant coverage (Wei et al., 2013; Arbabshirani et al., 2017).

The recent developments have indicated that along with the host's genetics, gut microbiota plays a very important role in the establishment of obesity and T2D (Karlsson et al., 2013; Bhute et al., 2017; Sroka-Oleksiak et al., 2020). Over the past decade around the world, significant efforts have been given by various groups to define the structural and functional attributes of gut microbiota in T2D subjects to NGT to understand the disease progression (Bhute et al., 2017; Gaike et al., 2020). Most of these studies attempted to evaluate the differences in gut microbial members either between T2D and pre-T2D with NGT or between gut microbiome after the treatment of the disease. However, the deep study on predicting the most important influencing physiological factors and their association with gut microbes in disease states is incompletely explained while none from India have been reported. Nevertheless, this investigation attempted to make the following contributions:

1) Introduce the most effective machine learning (ML) methods for better T2D and NGT predictions, as well as the most critical physiological parameters for detecting the disease regardless of its geographical location.
2) Analyze the variations in core gut microbial members between Indian T2D and NGT, and discover the differentially abundant core gut microbial genera, as well as their relationship to key physiological parameters.
3) Identify the specific microbial genera for each group (T2D and NGT) as crucial indicators for disease prediction and diagnosis using established physiological measures.

## Materials and Methods

### Feature Selection Approached Based on Machine Learning Techniques (MLT) and Evaluates the Prediction Model

#### Data Collection

For this study, the relevant physiological records of a total of 441 patient samples (T2D: 224 and NGT: 217) were considered. Among them, 345 data were obtained from Chinese cohorts (Qin et al., 2012) and 96 data from European cohorts (Karlsson et al., 2013). The physiological parameters included in our study were age, gender, body mass index (BMI), fasting blood glucose (FBG), fasting insulin (FI), hemoglobin A1c (HbA1c), cholesterol (CHL), high-density lipoproteins (HDL), low-density lipoproteins (LDL), triglycerides (TG), and C-peptide (CP).

#### Preparation of Training, Testing, and Blind/Identification Dataset

From 441 patients' physiological parameters data, we randomly generated a training dataset (with 150 samples) to train a prediction model and a testing dataset (with 150 samples) to assess the performance and ability to discriminate between two different classes (T2D and NGT) (Barman et al., 2014). A known blind/identification dataset was produced from the remaining 141 samples, but they were treated as an unknown dataset to evaluate the effectiveness of our predictive model. Finally, we applied this forecasting model to data obtained in Kolkata, West Bengal, and the surrounding areas (see sample collection section) to evaluate its performance on real-world unknown datasets.

#### Feature Selection and MLT

Feature selection improves the discrimination ability of the prediction model to relieve the over-fitting problem and help to better understand the data by examining the importance of the features (Saeys et al., 2007). Here, we used the recursive feature elimination (RFE) algorithm (Chen and Jeong, 2007) as a feature selection method to find out what was the best physiological parameters that showed higher discrimination ability between two classes using the “caret” R package (Kuhn, 2008). Random forest (RF) (Svetnik et al., 2003) and support vector machine (SVM) (Statnikov et al., 2013) were used for the prediction of T2D and NGT based on the physiological data. The prediction models were built up using 10-fold cross-validation methods.

#### Performance Checking of the Prediction Model

The performance of the prediction model was evaluated using the testing and blind datasets. To evaluate the performance of the prediction, they were assessed *via* sensitivity (SEN), specificity (SPF), accuracy (ACC), precision (PRC), and F1-score values. All these statistical analyses were performed in R (R, version 3.6.3) with the packages “randomForest” (Liaw and Wiener, 2002), “rfUtilities” (Evans and Murphy, 2019), “caret” (Kuhn, 2008), “caTools” (Tuszynski and Tuszynski, 2007), “e1071” (Meyer et al., 2012), “verification” (Gilleland, 2015) and “pROC” (Robin et al., 2011).

### Amplicon-Based Metagenomic Analysis of T2D and NGT Samples From West Bengal

#### Sample Selection and Collection

The samples were selected as per suggestion from the doctors of the endocrine department of IPGMER and SSKM Hospital, Kolkata, India based on World Health Organization (WHO) criteria, and anthropometric measurements were done from 34 samples (17 NGT and 17 T2D) from West Bengal at IPGMER and SSKM Hospital. Only newly diagnosed cases of T2D in males of age group above 25 years and up to 55 years, willing to take participation, were included in our study. The patients, in the age group below 25 years and above 55 years, already diagnosed or treated with insulin, were excluded from this study. The physiological parameters of all these samples were measured in the Endocrinology Lab of IPGMER and SSKM Hospital. The FI and CP were measured using Siemens Immulite Insulin and C-Peptide Kit and other remaining physiological data such as BMI, FBG, CHL, HDL, LDL, and TGL were measured by normal testing procedure (Zhang et al., 2013). The protocol and the project were approved by the ethics committee at SSKM Hospital.

#### The DNA Extraction and Amplicon Metagenomic Sequencing

The metagenomic DNA was extracted from the patients' fecal samples by using PowerFecal DNA Isolation Kit (Mo Bio, Catalog No. 12830-50) following the manufacturer's instructions. The extracted metagenomic DNA was pooled for the amplification of hypervariable V3–V4 regions of the bacterial 16S rRNA gene and sequenced them using the Illumina MiSeq platform (2 × 300 bp paired-end). The raw paired-end primer trimmed sequences were provided by Eurofins, India. All raw metagenomic DNA sequences were submitted to SRA–NCBI database (Accession No. PRJNA486712).

#### Sequence Processing and Taxonomy Classification

All the raw fastq datasets were processed by the following sequence processing protocol (Dhal et al., 2020; Nayak et al., 2021). For all 16S rRNA amplicon gene sequences from each sample, the quality screening was done by using Trimmomatic, version 0.33 (parameters: SLIDINGWINDOW: 4:15) (Bolger et al., 2014). High-quality sequence reads were then merged with PEAR, version 0.9.5 (Zhang et al., 2014), using default parameters. For operational taxonomy unit (OTU) clustering, SWARM, version 2.0, was used with default parameters (Mahé et al., 2014). Moreover, SINA tool was used for alignment and taxonomic classification using the SILVA ribosomal RNA gene database, version 138, as a reference sequence using the representative sequence per OTU (Pruesse et al., 2012). Absolute singletons OTUs, as well as unclassified sequences on phylum level, were removed from our dataset using our standardized R script.

#### Statistical Analysis

Principal component analysis (PCA) was done to understand the pattern among the two groups (T2D and NGT) of samples by utilizing their respective physiological data. To compare the physiological data of T2D and NGT groups, we used the Kruskal–Wallis rank–sum test.

Alpha (α) diversity analysis was done based on the rarefied data (minimum number of sequences among the samples) by sub-sampling the dataset. To assess the microbial communities' richness and evenness, OTU number (nOTU), inverse Simpson (invS), and Shannon diversity (shannon) were measured. The differences in α diversity between T2D and NGT were assessed by Wilcoxon rank–sum test. The unique and core bacterial members among the two groups (T2D and NGT) were identified by using Venny, version 2.1 (Oliveros, 2007), with genera that had >0.5% abundance. Spearman rank correlation was calculated to assess if there were any relationship between alpha-diversity and the physiological parameters and to identify the association between the physiological parameters and microbial genera.

For beta (β) diversity, OTUs data were pruned to exclude the rare biosphere by retaining OTUs that were present in one or more than one sequence in three or more than three samples. This reduction of the datasets did not change β diversity patterns (Mantel test; *r* > 0.9, *p* = 0.001). To test the differences in community-level (β diversity) among T2D and NGT groups permutational multivariate analysis of variance (PERMANOVA) was calculated. The contribution of physiological parameters for explaining the variation in community structure redundancy analysis (RDA) was calculated based on their centered log-transformed of pruned data using aldex.clr function with a median of 128 Monte Carlo Dirichlet of ALDEx2 R package. Forward model selection was carried out to assess which are the best physiological parameters to explain this variation in the community based on maximum adjusted R2 and minimum Akaike Information Criterion (AIC). The differentially abundant OTUs among the T2D and NGT groups were identified by using Dotplot. All statistical analyses, as well as figure visualizations, were performed in R, version 3.6.3, with the packages “vegan” (Oksanen et al., 2013) and “ALDEx2” (Fernandes et al., 2014), and the PCA plot was made using OriginPro 2021 software, version 9.8.0.200.

#### Co-Occurrence Network Analysis

The co-occurrence network analysis was performed to assess the complexity of the microbiome and identify potential keystone taxa for each group. The co-occurrence network was constructed with the OTUs that were present in 10% of samples and had more than 10 sequences for each group. We used Spearman's rank correlation to assess the association among microbial OTUs from each group. Moreover, *p* = ≤0.05 and a Spearman's rank correlation coefficient, ρ = ≥0.6 were selected as the thresholds between two OTUs (Jiao et al., 2016; Li et al., 2021). Two co-occurrence networks were built, the T2D co-occurrence network (TCN), and NGT co-occurrence network (NCN). The network's topology was measured by calculating the nodes, edges, average weighted degree, network diameter, graph density, modularity, average clustering coefficient, and average path length for each network. The network visualization and topology analysis were performed in the Gephi 0.9.2 (https://gephi.org/) visualization tool (Bastian et al., 2009). The role of nodes in individual co-occurrence network topology was determined by evaluating the within-module connectivity (Zi) and among-module connectivity (Pi) using a web-based tool, molecular ecological network analysis pipeline (MENAP) (http://ieg4.rccc.ou.edu/mena) (Deng et al., 2012; Qiu et al., 2022). Based on this analysis, the nodes are classified into the following four groups: (a) Peripheral nodes (Zi < 2.5, Pi < 0.62), (b) connectors (Zi < 2.5, Pi > 0.62), (c) module hubs (Zi > 2.5, Pi < 0.62), and (d) network hubs (Zi > 2.5, Pi > 0.62) (Qiu et al., 2022). The module hubs are densely connected to many nodes within *r* own modules, whereas the network hubs serve as both connectors and module hubs. Together with network hubs, module hubs, and connectors were termed an keystone nodes/taxa (Olesen et al., 2007; Zhou et al., 2010; Deng et al., 2012; Qiu et al., 2022).

## Results

### Physiological Parameters of Indian T2D and NGT Samples

**Figure 1:**
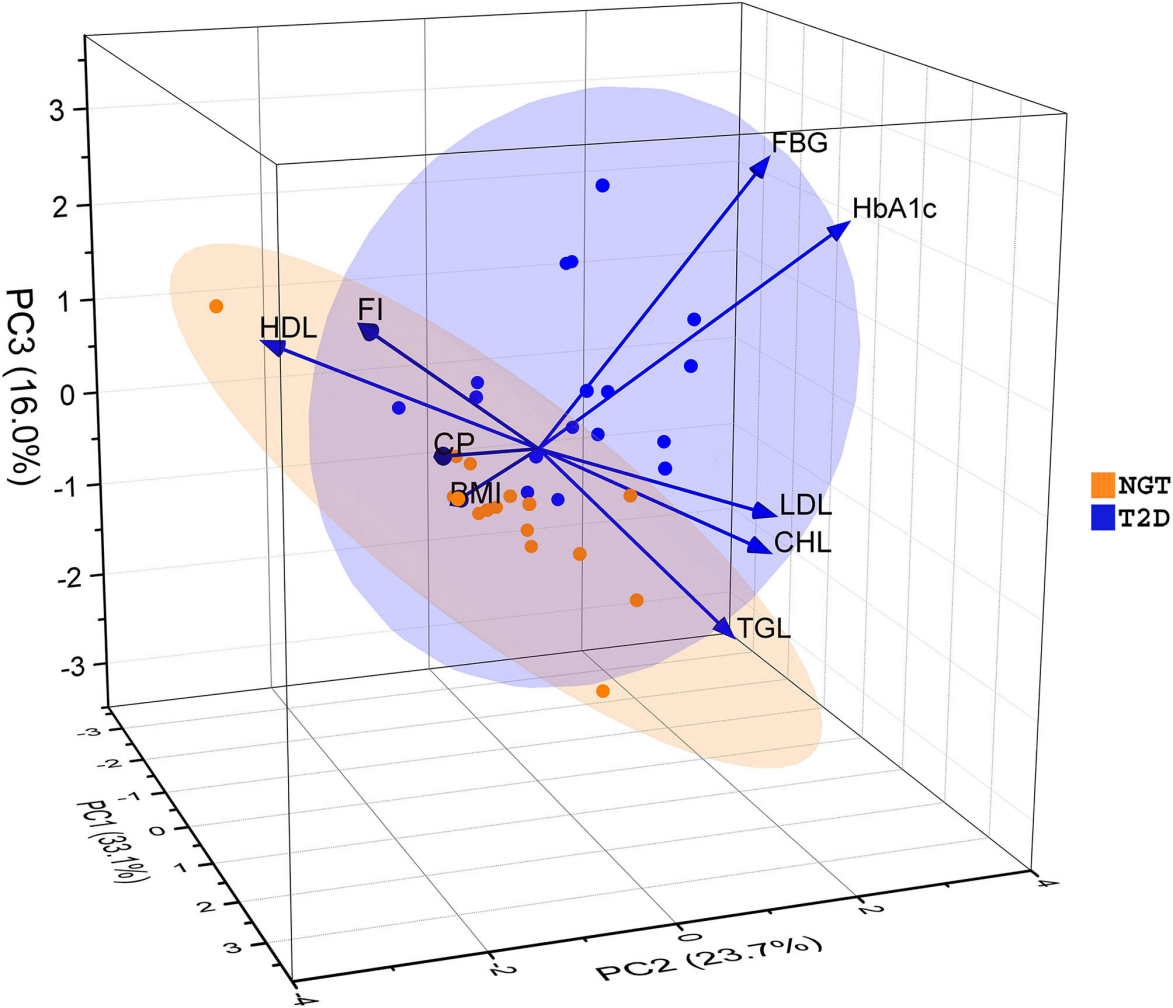
Principal Component Analysis (PCA) based on physiological parameters of the Indian diabetes subjects and controls. The samples were divided into two groups along with three principal components (PCs). PC1, PC2, and PC3 explained 33.1, 23.7, and 16 percent of the total variation respectively. Here BMI, Body Mass Index; FBG, Fasting Blood Glucose; FI, Fasting Insulin; HbA1c, Glycated Hemoglobin; CP, C – Peptide; CHL, Cholesterol; HDL, High-Density Lipoprotein; LDL, Low-Density Lipoprotein; TGL, Triglyceride.

The pathophysiological conditions of diabetes patients were assessed *via* nine different parameters (BMI, FBG, FI, HbA1c, CP, CHL, HDL, LDL, and TGL) of T2D with respect to NGT (Supplementary Table S1). Among them, the average level of FBG and HbA1c in the T2D group (168 mg/dl and 8.1% respectively) were found significantly higher (*p* ≤ 0.05) than NGT (Table 1). The PCA analysis indicates first three principal components accounted for 72.8% variation among the two groups of samples based on their measured physiological parameters (Figure 1). The PC1 alone explained 33.1% variation, majorly contributed by BMI, CP, CHL, and LDL; PC2 explained 23.7% of the total variation that was mainly driven by FBG, HbA1c, and TGL; and PC3 was responsible for the remaining 16% variation explained by FI and HDL. It was also evident that the T2D group was separated as a single cluster from the NGT group along the FBG and HbA1c parameters.

**Table 1:** Differences in physiological parameters between diabetes subjects and controls assess by Kruskal–Wallis rank–sum test.

**Parameters**	**χ^2^**	**DF**	***p***
Body Mass Index (BMI)	0.001	1	0.9725
Fasting Blood Glucose (FBG)	11.640	1	0.0006*
Fasting Insulin (FI)	0.050	1	0.8228
Glycated hemoglobin (HbA1c)	13.233	1	0.0003*
C – Peptide (CP)	0.015	1	0.9040
Cholesterol (CHL)	0.323	1	0.5698
High Density Lipoprotein (HDL)	1.909	1	0.1671
Low Density Lipoprotein (LDL)	0.001	1	0.9725
Triglycerides (TGL)	0.058	1	0.8094

^*^Indicates highly significant.

### Selection of Optimal Features, Construction, and Performance Evaluation of MLT Models to Classify Between T2D and NGT

Feature selection (FS) is a pattern recognition application to remove the irrelevant or noise from the original features data. The RFE FS is a multivariate approach that incorporates all variables in the algorithm and gradually excludes those variables which are not able to discriminate between the different classes. In this study, nine physiological parameters (BMI, FBG, HbA1c, FI, CP, CHL, HDL, LDL, and TGL) of a total of 441 samples were considered to identify the best physiological parameters having the discriminatory ability between T2D and NGT and we have found five best physiological parameters (through RFE FS) that includes FBG, HbA1c, CP, FI, and CHL with high accuracy (ACC = 95%).

For this investigation, those five important physiological parameters were further used to build as well as to evaluate the performance of the prediction models using three different MLT methods, i.e., RF, SVM–L, and SVM–R. The prediction models were built with 150 training datasets (75 T2D and 75 NGT) and performance of these prediction models were tested using the same number of the testing datasets (75 T2D and 75 NGT) by measuring their SEN, SPF, ACC, and PRC with 10-fold cross-validation. However, the best prediction models were measured by their performance checking of precision (PRC) and recall (also known as SEN) since they were directly proportional to the true positive (Barman et al., 2014). All the prediction models worked very well and the values of SEN, SPF, and ACC of the three prediction models were nearly the same. However, the PRC score in SVM–L (100%) was higher than RF (94%) and SVM–R (94%), while the recall score of RF (100%) was higher than the SVM–L and SVM–R (Table 2). However, they were further evaluated to confirm their discriminatory abilities between T2D and NGT using a blind dataset.

**Table 2:** Comparative performance measurement among three different MLT methods using three different datasets with 10-fold cross-validation.

**Datasets**	**MLT**	**Sensitivity**	**Specificity**	**Accuracy**	**Precision**
Test dataset	RF	1.00	0.98	0.97	0.94
	SVM–L	0.97	1.00	0.98	1.00
	SVM–R	0.98	0.94	0.96	0.94
Blind dataset	RF	1.00	0.88	0.94	0.90
	SVM–L	0.81	0.97	0.88	0.96
	SVM–R	1.00	0.88	0.94	0.90
Unknown dataset	RF	1.00	0.52	0.76	0.68
	SVM–R	1.00	0.35	0.67	0.60

*RF, Random forest; SVM–L, Support vector machine with linear Kernel; SVM–R, Support vector machine with RBF Kernel*.

### Evaluation of Prediction Methods With Blind Dataset and Classification of Unknown Samples

We used the same approach to avoid any bias in the performance of our proposed models and observed how well they could distinguish between the two classes. Our analysis reported that all three prediction models worked very well to classify the T2D and NGT blind. Both RF and SVM–R models were able to identify the total 74 T2D samples correctly, (100% SEN values) while SVM–L showed the best prediction efficiency (97% SPF value) compared to the other two (Table 2). Overall, this investigation reported that the best two effective prediction models are random forest (RF) and SVM–R (SVM with RBF Kernel) as indicated on precision (PRC) and recall (SEN) values.

The collected physiological parameters of 34 samples (17 T2D and 17 NGT), as unknown datasets, were used to further evaluate the efficiency of RF and SVM–R prediction models using the top-five physiological data that were identified in RFE–FS. Both prediction models were successful in classifying all T2D samples as a true positive with 100% SEN or recall (Table 2). Interestingly, from the above study, it is observed that FBG and HbA1c were demonstrated as the most important discriminative parameters with the highest mean decrease scores (95.2 and 75.2%, respectively) among the two study groups.

### Diversity Analysis and Taxonomy Composition of the Indian T2D and NGT

By removing primer sequences of microbial hypervariable V3–V4 region of 16S rRNA gene amplicon sequences, a total of 71,30,226 clipped pair-end reads were generated. After trimming and merging the paired-end reads, a total of 44,00,731 merged sequences were obtained (Supplementary Table S2). The high-quality reads were then clustered using > 97% sequence identity which generated 7,71,043 OTUs. A total of 43,467 swarm OTUs was obtained by removing the absolute singletons and unclassified sequence at the phylum level to avoid the rare biosphere, potential chimera effects, and PCR artifact (Dhal et al., 2020; Nayak et al., 2021).

**Figure 2:**
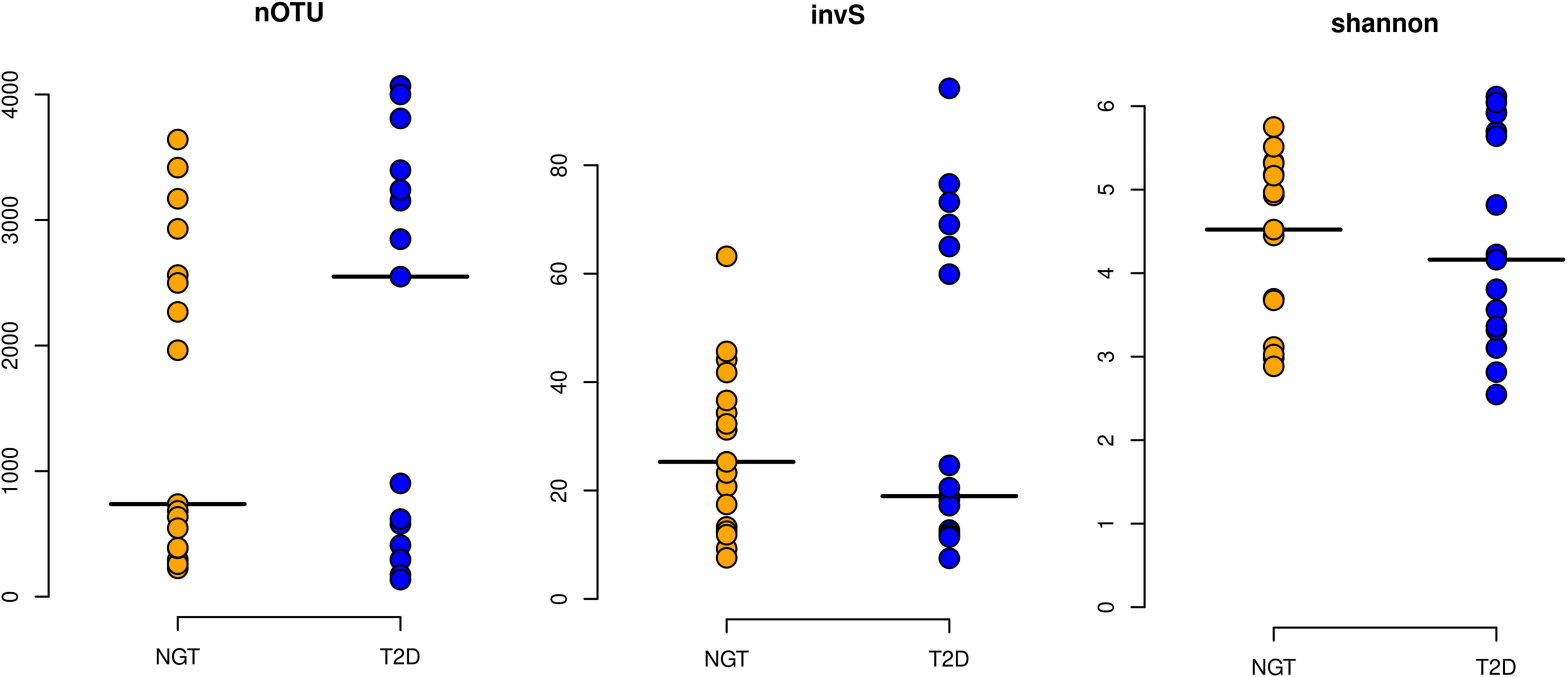
Alpha diversity Indices. The alpha diversity of the studied groups was measured based on their richness [nOTU and inverse Simpson index (invS)] and evenness [Shannon Index (shannon)]. Here horizontal lines in the plot represent their respective mean value.

α diversity i.e., diversity within the sample, was measured through nOTUs, Shannon diversity index as well as inverse Simpson index. It was observed that the average nOTU was higher in the T2D group (1960) than in the NGT (1565). Similar results were observed for Species richness and evenness in T2D and NGT groups as indicated by the Shannon diversity and inverse Simpson index (Figure 2). Spearman rank correlations test indicated a strong association of FBG with alpha diversity of the T2D group (ρ = 0.54, *p*-value ≤ 0.05) but none in NGT.

**Figure 3:**
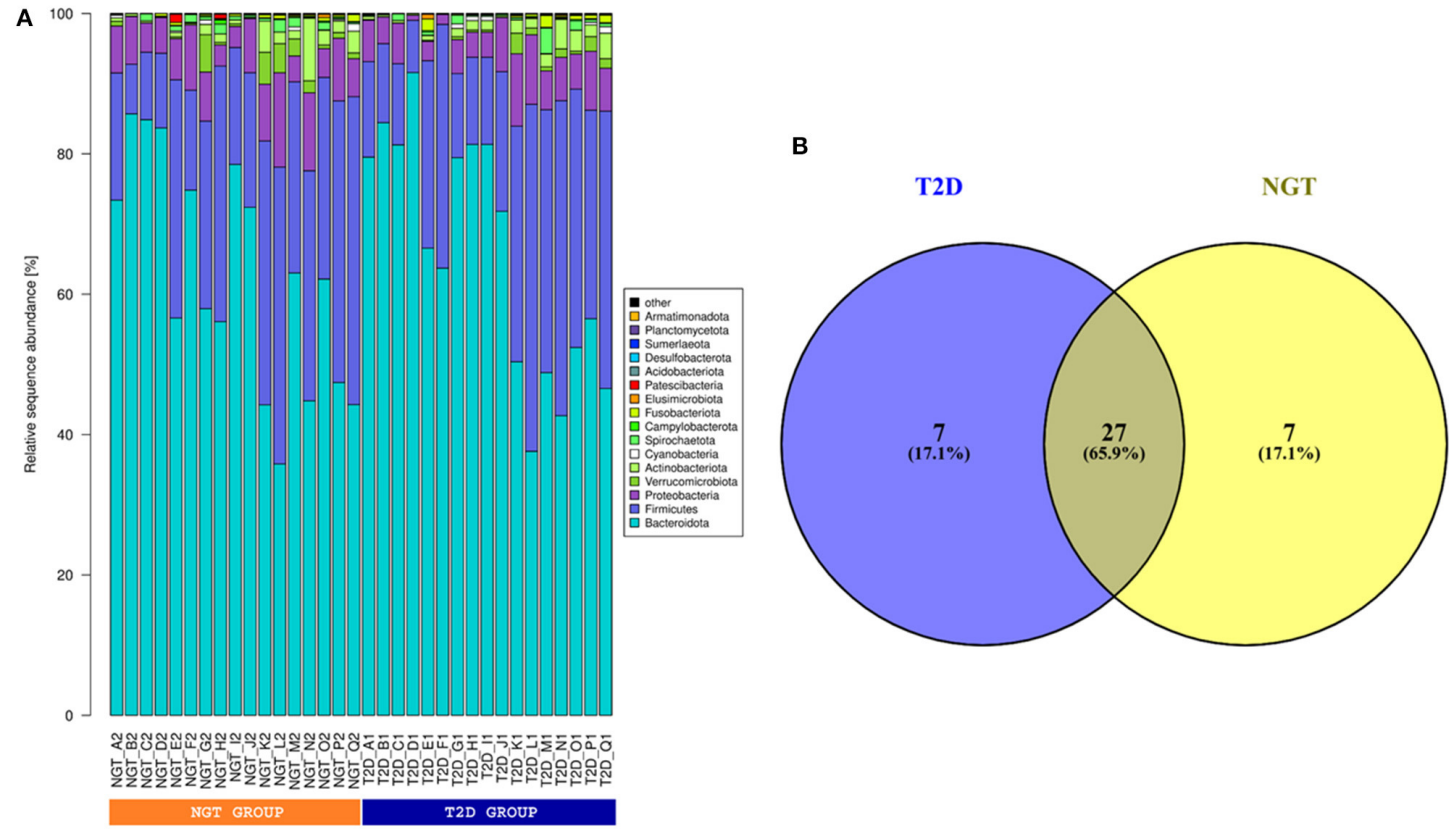
**(A)** Phylum level taxonomic composition. Relative sequence abundance of most (top 10 based) dominant gut microbes in phylum level of studied samples. **(B)** Venn diagram for unique and common gut microbes. According to the Venn diagram, 27 gut microbes were common for both T2D and NGT groups, and 7 and 7 gut microbes were unique for T2D and NGT groups respectively.

The bacterial communities of gut microbiota were dominated by the members of *Bacteroidota, Firmicutes, Proteobacteria*, and *Actinobacteria* which represented almost 97% of sequences (Figure 3A). In this study, we also observed 27 bacterial genera representing the core gut microbiome in the studied samples while each of 7 bacterial genera was found as unique for the T2D and NGT microbiome (Figure 3B). The core microbiome was mainly dominated by *Prevotella_9, Prevotella, Prevotellaceae Incertae Sedis, Bacteroides*, and *Alloprevotella* of *Bacteroidia*; *Lachnospiraceae Incertae Sedis, Roseburia*, and *Faecalibacterium* of *Clostridia*; *Megasphaera* of *Negativicutes* and *Succinivibrio* of *Gammaproteobacteria* (Supplementary Figure S1, Supplementary Table S3). The unique bacterial member for the T2D microbiome was composed of *Eubacterium eligens group, Lachnoclostridium, Ruminococcus torques group*, and *Clostridia vadinBB60 group Incertae Sedis*, and *Lachnospira* under the class *Clostridia*; *Haemophilus* of *Gammaproteobacteria* and *Catenibacterium* of *Bacilli* (Supplementary Table S5). While *Alistipes* and *Muribaculaceae Incertae Sedis* under the class *Bacteroidia*; *Ligilactobacillus* and *Holdemanella* of *Bacilli*; *Enterobacter* of *Gammaproteobacteria*; *Blautia* and *Coprococcus* of *Clostridia* were observed only in the NGT group (Supplementary Table S4).

Also, β diversity was a measure to determine the intra-sample variation of the gut microbial community using the pruned 6903 OTU datasets. The differential OTUs using the ALDEx2 test reported a total of 61 OTUs representing 68.1% of total communities for T2D and NGT gut microbiome that include classes *Bacteroidia* (34 OTUs), *Clostridia* (13 OTUs), *Gammaproteobacteria* (5 OTUs), *Negativicutes* (4 OTUs), *Spirochaetia* (2 OTUs), *Bacilli* (2 OTUs), and *Verrucomicrobiae* (1 OTU), which were deferred as differential abundant between T2D and NGT (Supplementary Figure S2).

Within *Bacteroidia*, OTU affiliated with genus *Prevotella_9* (15 OTUs), *Alloprevotella* (otu18 and otu36), *Bacteroides* (otu28), *Prevotella Incertae Sedis* (otu48), and *Rikenellaceae RC-9 gut group* (otu82) significantly enriched in the T2D microbiome whereas *Prevotella* (otu22, otu24, and otu116) significant enriched in NGT microbiome. Within the Clostridia class, *Eubacterium* (otu49 and otu59) and *UCG-002* (otu46) genera were found dominant in the T2D microbiome, whereas *Roseburia* (otu38 and otu51), *Lachnospiraceae Incertae Sedis* (otu43 and otu112), *Butyrivibrio* (otu55), and *Faecalibacterium* (otu42) genera were found significantly enriched in NGT microbiome. Similarly, *Gammaproteobacteria, Haemophilus* (otu237) showed dominance in the T2D microbiome whereas *Klebsiella* (otu83) and *Succinivibrio* (otu17) genera were found highly enriched in NGT. It was also observed that within *Negativicutes* genera, *Phascolarctobacterium* (otu33) was significantly dominant in the T2D microbiome, but in the same class, *Megasphaera* (otu25) and *Selenomonadaceae Incertae Sedis* (otu150) genera were significantly dominant in the NGT microbiome. Within *Bacilli*, the genus *Asteroleplasma* (otu64) significantly enriched in the T2D group whereas under the class *Spirochaetia* and *Verrucomicrobiae, Treponema* (otu81 and otu104), and *Akkermansia* (otu100) genera showed most dominance in the NGT group, respectively.

**Figure 4:**
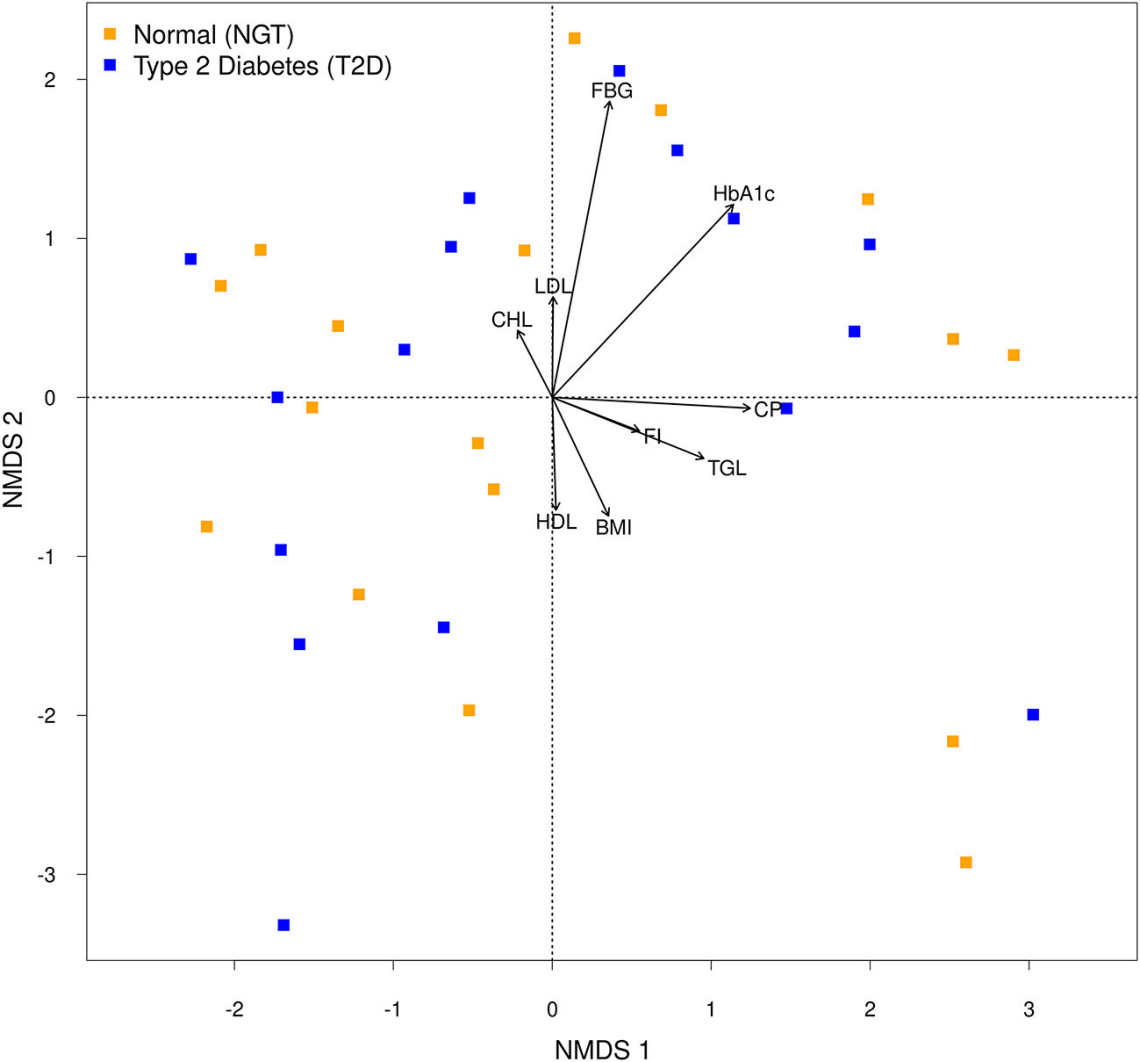
Non-metric multidimensional scaling (NMDS) plot of the bacterial communities of each group. Arrows of the NMDS plot indicate *envfit* correlations of bacterial community composition with physiological parameters.

Similarities or dissimilarities between two groups were projected in an ordination space as well as their associated physiological parameters on the NMDS plot (Figure 4). Moreover, *Envfit* result showed that FBG (R^2^ = 0.2022, *p* = 0.025) and HbA1c (R^2^ = 0.1480, *p* = 0.086) coincided with microbial community composition, but the association seems to be weak. Redundancy analysis which was performed to assess the significant contribution of the tested parameters in describing the variation in microbial communities revealed that only HbA1c had the explanatory power for bacterial communities of T2D microbiota with 2.1% (Adj. R^2^ = 0.021, F = 1.34, AIC = 168.51, *p* = 0.05). Together NMDS and RDA supported each other's results and suggested that HbA1c, as well as FBG, were the responsible variable among the parameters for variation in the microbial composition in the T2D group.

**Figure 5:**
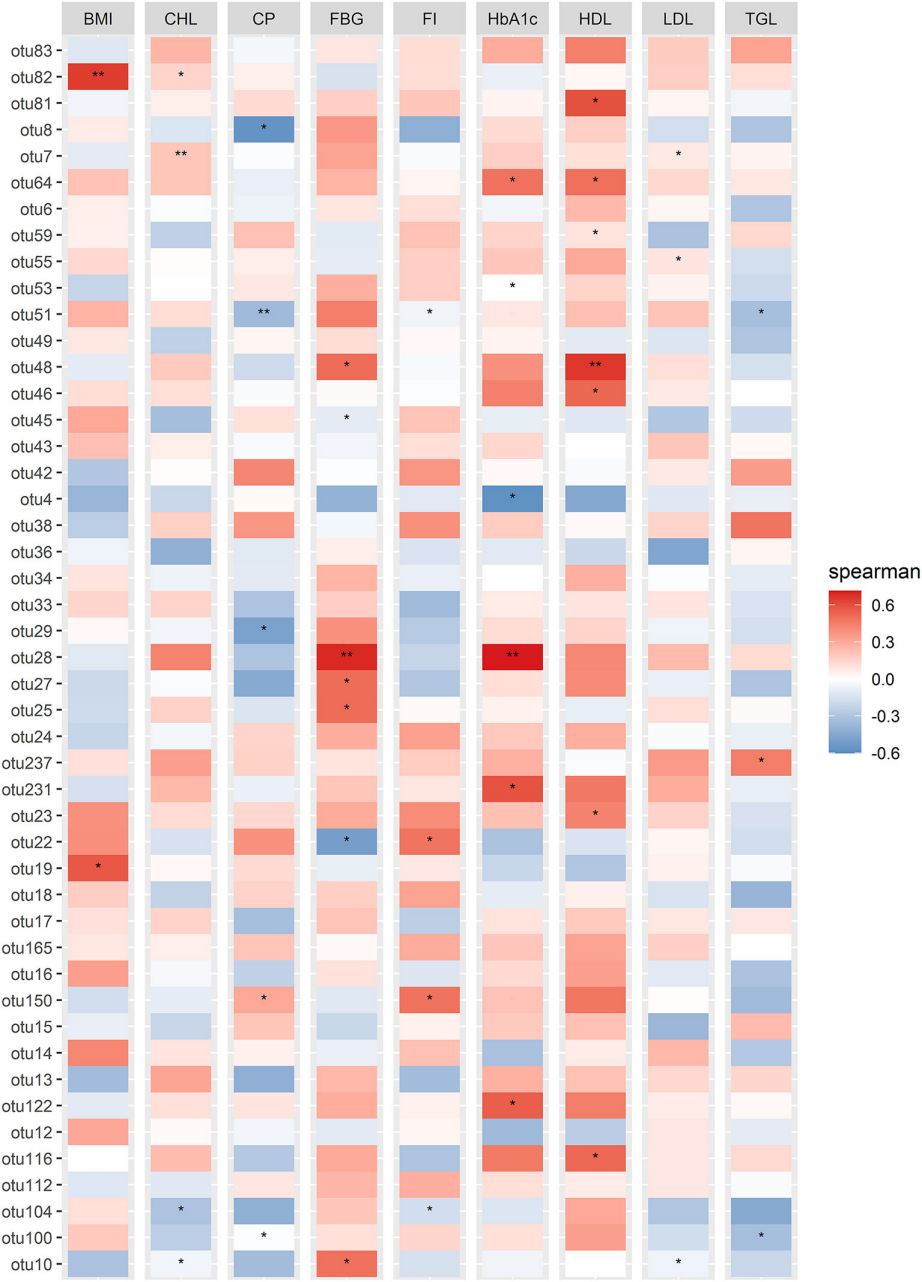
Correlation Heatmap of physiological parameters with the significant differential abundant OTUs identified in DotPlot analysis. Spearman correlation analysis based on differentially abundant significant OTUs and the measured physiological parameters. Spearman correlation values were shown in the vertical heatmap panel to the right. *P* ≤ 0.05 were indicated by the “*” symbols.

The significant correlation between the significant differentially abundant OTUs with the most important physiological parameters (FBG and HbA1c, as they were found as the most significant influence in our statistical analysis) was measured by calculating the Spearman correlation coefficient (SCC). As indicated in Figure 5, otu10, otu27, and otu231 represent *Prevotella_9*, otu28 represent the *Bacteroidandes*, otu48 represent the *Prevotella Incertae Sedis* showed a significantly positive correlation with FBG (*p* ≤ 0.05) while out53, otu122, and otu231 representing *Prevotella_9*, otu64 representing *Asteroleplasma* and otu28 representing *Bacteroides* were highly positively correlated with the HbA1c (*p* ≤ 0.05).

### Co-Occurrence Network Analysis and Keystone Taxa of the Indian T2D and NGT

**Figure 6:**
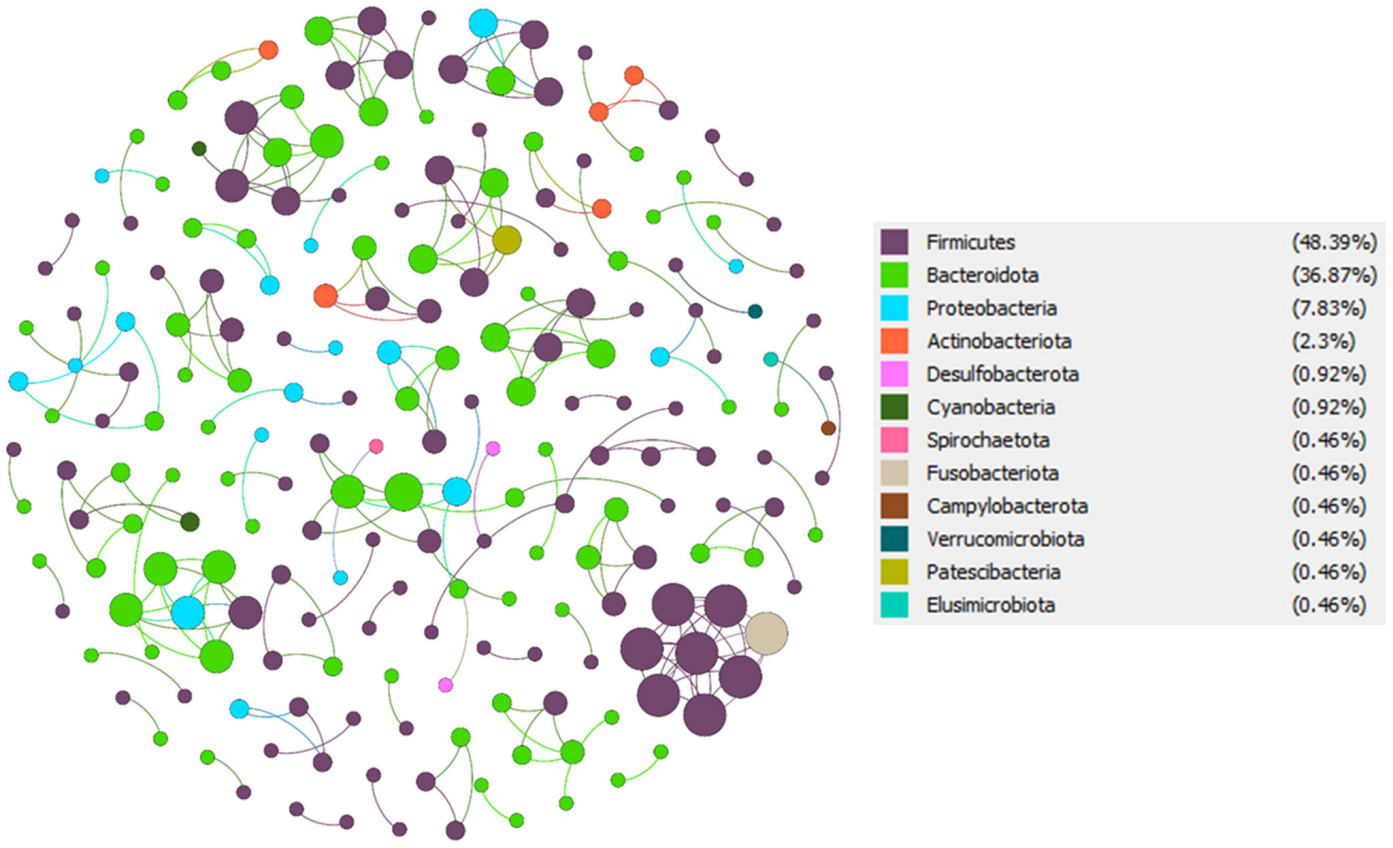
NGT co-occurrence network (NCN). From total OTU abundance data, we select the NGT specific OTUs using the specified criteria, and a co-occurrence microbial network was constructed in Gephi.

**Figure 7:**
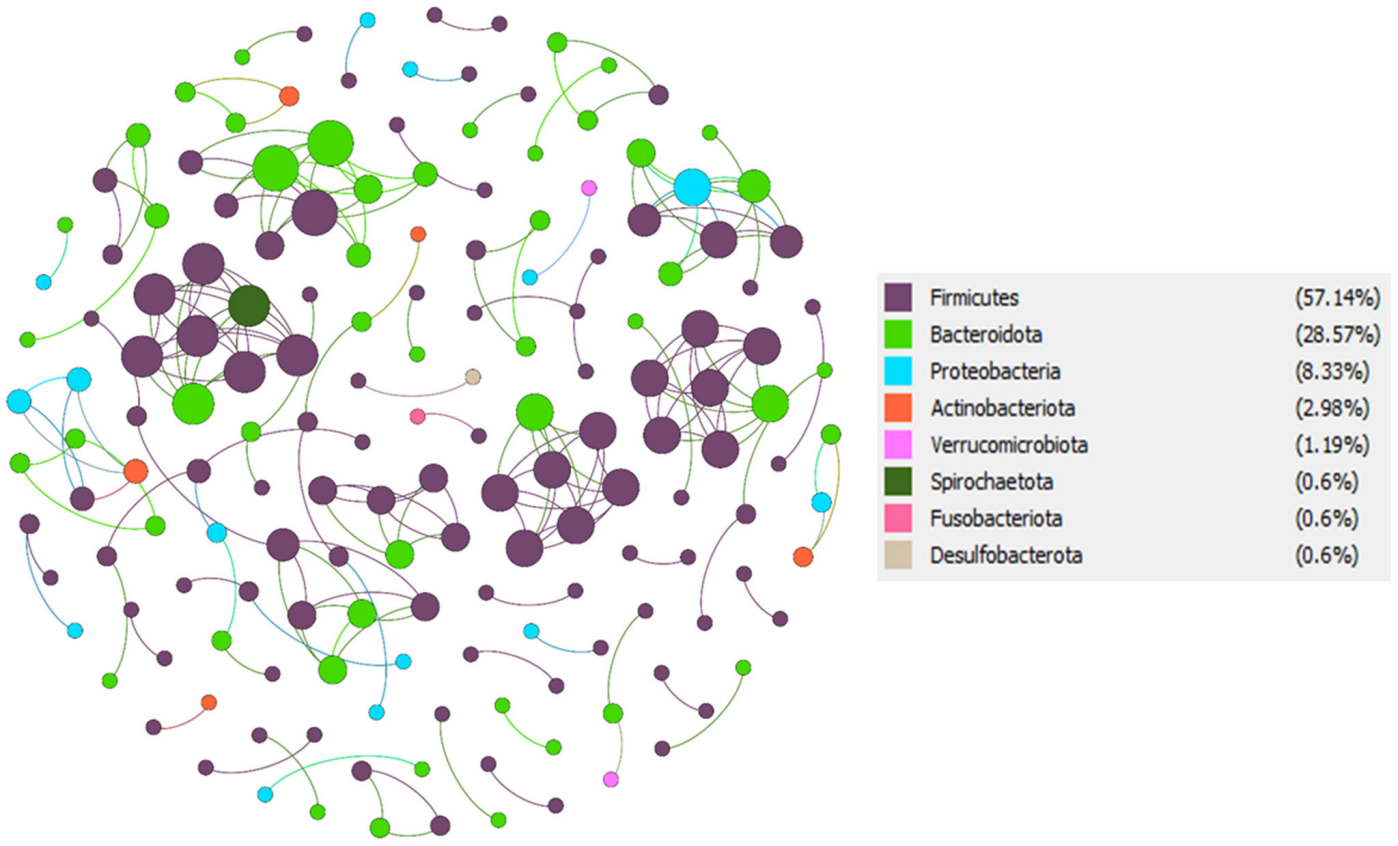
T2D co-occurrence network (TCN). From total OTU abundance data, we select the T2D specific OTUs using the specified criteria, and a co-occurrence microbial network was constructed in Gephi.

To understand the potential interactions among gut microbial community members for each group, we constructed co-occurrence networks based on OTU to OTU correlations. The T2D co-occurrence network (TCN) consisted of 168 nodes and 213 edges, while the NGT co-occurrence network (NCN) consisted of 217 nodes and 233 edges (Table 3). The modularity of TCN is 0.93 decreased from NCN modularity (0.96), accompanying the increase of average weighted degree in TCN (1.268) compared to NCN (1.074). The nodes present in both TCN and NCN networks were mostly dominated by phyla *Firmicutes, Bacteroidota, Proteobacteriota, Verrucomicrobiota, Spirochaetota, Fusobacteriota*, and *Desulfobacterota* (Figures 6, 7). However, their percentage in each network was different, such as the *Firmicutes* present in TCN and NCN is 57.14 and 48.39%, respectively; the same trend was also observed in *Bacteroidota* (TCN *vs*. NCN: 28.57 *vs*. 36.87%), *Proteobacteria* (TCN *vs*. NCN: 8.33% *vs*. 7), *Actinobacteria* (TCN *vs*. NCN: 2.98 *vs*. 2.3%), *Verrucomicrobiota* (TCN *vs*. NCN: 1.19 *vs*. 0.46%), *Spirochaetota* (TCN *vs*. NCN: 0.6 *vs*. 0.46%), *Fusobacteriota* (TCN *vs*. NCN: 0.6 *vs*. 0.46%), and *Desulfobacterota* (TCN *vs*. NCN: 0.6 *vs*. 0.92%). *Cyanobacteria* (0.92%), *Campylobacterota* (0.46%), *Patescibacteria* (0.46%), and *Elusimicrobiota* (0.46%) gut microbial phyla were found only in the NCN, while none from TCN. We also identified 14 and 8 OTUs as keystone nodes from TCN and NCN networks, respectively, based on within-module connectivity (Zi) and among-module connectivity (Pi) values. Among them, six OTUs as module hubs and eight OTUs as connector nodes were identified in the TCN network, whereas in the NCN network, seven OTUs as module hubs and one OTU as connector node were identified. The identified keystone taxa, five OTUs were found under the phylum *Firmicutes*, four for *Bacteroidota*, three for *Proteobacteria*, one for *Actinobacteriota*, and one for *Spirochaetota* gut microbial phyla in TCN network. In contrast, two OTUs were found under the phylum *Bacteroidota*, three for *Firmicutes*, one for *Proteobacteria*, one for *Patescibacteria* and one for *Desulfobacterota* as keystone microbial phyla for NCN. Due to the decrease in network topology and different gut microbial compositions, the network stability also decreases in TCN compared to NCN.

**Table 3:** Characteristics information of two gut microbial co-occurrence network; TCN–T2D co-occurrence network, NCN–NGT co-occurrence network.

**Network Topology Parameters**	**NCN**	**TCN**
Number of nodes	217	168
Number of edges	233	213
Average weighted degree	1.074	1.268
Network diameter	3	2
Graph density	0.005	0.008
Modularity	0.96	0.93
Average clustering co-efficient	0.226	0.208
Average path length	1.084	1.082

## Discussion

Many reports endorsed the usefulness of different machine learning techniques to discriminate between T2D and NGT using a patient's physiological conditions, but none has attempted to identify the important parameters that can alone predict and diagnose the T2D (Choi et al., 2019; Tigga and Garg, 2020). In this study, we are the first to attempt to develop an MLT-based prediction model using the conventional classification algorithms as well as identification of the most important physiological parameters (using the feature selection method, RFE) to classify diabetes status. Our prediction models are developed and verified using two different regions of datasets (Chinese and European) and applied these models to the studied Indian samples, to avoid any geographic biases. Our proposed prediction models, RF and SVM with RBF Kernel (SVM–R) have outperformed other already established models with high accuracy (94%) (Choi et al., 2019). Those models also identify the two most important physiological parameters, FBG and HbA1c, which have a greater role in the classification of T2D and diagnosis of the disease which is in line with the American Diabetes Association (ADA) and the World Health Organization (WHO) recommendations as well as previous investigations, stating that both FBG and glycated hemoglobin (HbA1c) are critical to classify the T2D patients (Chaudhury et al., 2017; Deberneh and Kim, 2021).

Our statistical analysis also supports the result of MLT analysis by showing significant differences among FBG and HbA1c levels of the studied Indian T2D when compared to NGT, which have also separately ordinate from each other along with those parameters in the PCA plot. So, the significant changes in the level of both FBG and HbA1c can be used as critical physiological measurements to identify the T2D patients or risk of disease in impaired states of patients around the world.

Alterations of gut microbiota and their association with T2D are well-established around the world (Karlsson et al., 2013; Bhute et al., 2017; Gaike et al., 2020; Sroka-Oleksiak et al., 2020). However, the microbial dynamism of T2D patients from normal as well as their correlation with the important physiological parameters (FBG and HbA1c) is not reported, which is another novelty of our investigation. In this study, we were the first to provide the preliminary information on the gut microbiome of T2D patients from the eastern region of the Indian Subcontinent, especially in and around Kolkata, West Bengal. The T2D patients from this region have unique dietary status compared to other regions and this seems to restrict us from collecting the samples from different regions which is also reflected in our sample size. The microbial community of the studied samples was dominated by the members of the bacterial groups under phylum *Bacteroidota, Firmicutes, Proteobacteria*, and *Actinobacteria*. *Bacteroidota* and *Firmicutes* are the well-known dominant bacteria phylum found in obesity, diabetes, and also in normal gut microbiome around the world (Gaike et al., 2020; Sroka-Oleksiak et al., 2020). Although there are reports on the differences in abundance among *Bacteroidota* and *Firmicutes* in T2D patients to NGT (Zhang et al., 2013; Ahmad et al., 2019). However, some other reports stated that such differences are not significant in T2D from NGT, which is in line with our results, as this investigation mostly focused on T2D irrespective of their obesity status (Turnbaugh et al., 2006; Ley et al., 2008; Zhang et al., 2013). The members of phyla *Firmicutes* play an important key role in fat digestion and their higher abundance is directly associated with obesity whereas *Bacteroidota* is associated with the production of short-chain fatty acids (SCFAs) (Ahmad et al., 2019).

Among the 27 core bacterial genera, the taxonomy of the associated genera with significantly dominated OTUs in studied T2D samples is *Prevotella_9, Alloprevotella, Bacteroides, Prevotella Incertae Sedis, Rikenellaceae RC-9 gut group, Eubacterium, UCG-002, Phascolarctobacterium*, and *Asteroleplasma*. They are also reported to be well-associated with T2D; for example, *Allopreotella* and *Bacteroides* are reported as risk factors for diabetes as these are reported to increase the level of lipopolysaccharides (LPS) and insulin resistance, which are detrimental to human health (Cheng et al., 2017; Wang et al., 2020). The *Prevotella_9* is reported to be associated with a plant-based low-fat diet and represents key bacterial members during human gut microbiota maturation in infants to young adults (Qian et al., 2018; Li et al., 2020b). However, the biological significance in the human gut enterocyte of both *Prevotella_9* and *Asteroleplasma* has not been well elucidated. While *Rikenellaceae RC9 gut group* bacterial genera showed an association with a high-fat diet and play an important role in lipid metabolism (Zhao et al., 2018). The genus *Phascolarctobacterium* is reported as an enriched bacterial genus in the T2D mice model and negatively correlated with fasting insulin (Naderpoor et al., 2019; Song et al., 2020). We found OTUs representing *Prevotella_9, Bacteroides, Prevotella Incertae Sedis* and *Asteroleplasma* bacterial genera have a significantly positive correlation with important established physiological parameters FBG and HbA1c. Interestingly, this observation supported the correlation analysis of alpha-diversity (richness and evenness) of the gut microbial community of studied T2D patients with FBG. Also, the results of NMDS *envfit* and RDA reflect that FBG and HbA1c both coincided most strongly with the microbial community composition of the T2D microbiome. On the other hand, *Prevotella, Roseburia, Lachnospiraceae Incertae Sedis, Butyrivibrio, Faecalibacterium, Klebsiella, Succinivibrio, Megasphaera, Selenomonadaceae Incertae Sedis, Treponema*, and *Akkermansia* genera are found as dominant bacterial genera in the NGT microbiome. A similar result was observed in the study by Almugadam et al. (2020) where they reported that short-chain fatty acid (SCFA) and butyrate producers such as *Faecalibacterium, Roseburia, Selenomonadaceae Incertae Sedis, Succinivibrio*, and *Megasphaera* genera were abundant in the healthy gut microbiome (Almugadam et al., 2020). *Prevotella, Succinivibrio, Treponema*, and *Lachnospiraceae* Incertae Sedis major contributes to inter-individual variation in gut microflora and are associated with better digestion of plant-derived complex carbohydrates and fibers diet for glucose homeostasis along with the production of butyric acid in the human colon for intestinal barrier protection (Arumugam et al., 2011; Schnorr et al., 2014; De Filippo et al., 2017; Zhao et al., 2020). Several investigators report the enrichment of butyrate-producing bacterial genera such as *Roseburia, Butyrivibrio, Faecalibacterium, Lachnospiraceae Incertae Sedis*, and *Megasphaera* are responsible for the reduction of inflammatory symptoms as well as insulin resistance. These bacterial genera play an important key role in intestinal health maintenance, immune defense, regulation of the dynamic balance of T-cells, and promote Treg cell differentiation by butyrate production (Canani et al., 2011; Karlsson et al., 2013). *Klebsiella* bacteria are also found in the healthy human intestines and are not reported to be pathogenic as long the person is sick because of pneumonia, bloodstream infections, wound, or surgical site infections, etc. (Canani et al., 2011). A high abundance of mucin degrading *Akkermansia* bacterial genus in healthy human guts is well documented as they play a vital role in insulin resistance as well as intestinal barrier and LPS leakage reduction (Tanca et al., 2017; Gurung et al., 2020). Although some recent reports indicate that a decrease in this genus in diabetes is associated with inflammation and metabolic disorders in the mice model, it can be used as a biomarker for impaired glucose tolerance (Sonnenburg and Bäckhed, 2016; Plovier et al., 2017).

Several unique bacterial genera are identified in T2D compared to the NGT microbiome and probably play some roles in the structural and functional attributes of the gut microbes in the human intestine for the development of disease. The unique genera for the T2D microbiome are *Catenibacterium, Eubacterium eligens group, Lachnoclostridium, Ruminococcus torques group, Clostridia vadinBB60 group Incertae Sedis, Lachnospira*, and *Haemophilus*. Several investigators reported that a few of these bacterial genera such as *Ruminococcus torques group, Lachnospira*, and *Haemophilus* act in mucus degradation by decreasing the gut barrier integrity, and they can be used as bacterial biomarkers to study their involvement in the human gut or their uses as diagnostic tools should be encouraged (Chen et al., 2020; Vacca et al., 2020). *Haemophilus* bacterial genus reported highly abundant in the Chinese T2D cohort is a particular biomarker for them (Chen et al., 2020). While for NGT, the unique bacterial genera are *Enterobacter, Ligilactobacillus, Alistipes, Muribaculaceae Incertae Sedis, Blautia, Holdemanella*, and *Coprococcus* identified in this investigation. Few of those genera including, *Alistipes, Blautia*, and *Holdemanella* are observed in the normal human gastrointestinal tract and they have an important key role in protection from many diseases such as liver and cardiovascular fibrotic disorders and also from various pathogens (Arumugam et al., 2011; Parker et al., 2020). *Coprococcus, Muribaculaceae Incertae Sedis*, and *Enterobacter* bacterial genera are having the ability for metabolic improvements and consorted with a higher quality of life indicators supported by previous reports (Valles-Colomer et al., 2019; Wang et al., 2020).

Our co-occurrence network analysis showed that in T2D disease condition, significant changes in microbial network topological properties leads to a decrease in network stability and alteration in the microbial community in the human gastrointestinal tract, which is also in line with previous studies where they were reported, network complexity of the gut microbial community association was decreased in T2D (Li et al., 2020a). Interestingly co-occurrence network analysis also revealed that there are significant differences present in the proportion of taxonomic abundance of *Firmicutes* and *Bacteroidota* phylum in T2D compared to the NGT group which is also in line with the previously reported data (Turnbaugh et al., 2006; Ley et al., 2008; Zhang et al., 2013; Ahmad et al., 2019). The same trend was also observed in identified keystone taxa from the two co-occurrence networks and they might play an essential role in maintaining the microbial structure links, information transmission, and ecological function of the entire ecological communities in the gastrointestinal tract (Li et al., 2020a,b, 2021).

This investigation gives a well-resolved picture of the bacterial diversity and their correlation with important physiological parameters that influence the decrease of SCFA and butyrate-producing core bacteria which are beneficial for the human gut in T2D patients, in West Bengal, India. Also, we suggest that along with the well-established physiological parameters, the unique gut microbes can be used as a key biomarker to improve the disease diagnosis.

The Indian population size is large and has diverse dietary compositions or food habits with large metabolic differences. Recently, one report on the gut microbiota of T2D from the western part of India (Maharashtra, especially, in and around the city, Pune); however, none are from other regions/parts of this country (Gaike et al., 2020). In this study, we were the first to provide the preliminary information on the gut microbiome of Indian T2D patients from the eastern region of the Indian Subcontinent, especially, in and around the Kolkata, West Bengal, with almost similar dietary status and this seems to restrict us from increasing the sample size. This is a preliminary dataset that will help us formulate strategies to collect more samples from a diverse population for a deep understanding of the gut microbiome in Indian T2D patients. With the increase in the sample size, we will be able to perform more in-depth microbial diversity analysis and learn more about what governs the distribution of gut microbial taxa and how these distributions, as well as their ecosystem contributions in Indian T2D patients, will help to improve more accurate diagnosis of T2D disease in the future.

## Conclusion

From the investigation in this study, following conclusions can be drawn:

1) Random forest (RF) and support vector machine with RBF Kernel (SVM–R) are the best prediction models to predict the T2D and normal state based on a patient's physiological condition.
2) Fasting blood glucose and HbA1c individually or together can be used for the T2D diagnosis as well as defining the disease in an impaired state. Also, both of these physiological parameters coincided with the microbial community composition of the T2D microbiome by decreasing the beneficiary core gut microbial members.
3) *Catenibacterium, Eubacterium eligens group, Lachnoclostridium, Ruminococcus torques group, Clostridia vadinBB60 group Incertae Sedis, Lachnospira*, and *Haemophilus* can be used as important biomarkers for Indian T2D patients.

## Data Availability Statement

The datasets presented in this study can be found in online repositories. The names of the repository/repositories and accession number(s) can be found below: https://www.ncbi.nlm.nih.gov/, PRJNA486712.

## Ethics Statement

The studies involving human participants were reviewed and approved by Ethical Committee at SSKM Hospital. The patients/participants provided their written informed consent to participate in this study.

## Author Contributions

Conceptualization and supervision: PD. Data-curation: DD and PD. Formal analysis and writing the original draft: DD, TN, and PD. Methodology: PD, DD, and SC. Writing-review and editing: PD, DD, TN, and SC. All authors contributed to the article and approved the submitted version.

## Funding

This work was supported by the UGC–BSR Start-up-grant and JU–RUSA 2.0, DST (Grant No.: R-11/446/19).

## Conflict of Interest

The authors declare that the research was conducted in the absence of any commercial or financial relationships that could be construed as a potential conflict of interest.

## Publisher's Note

All claims expressed in this article are solely those of the authors and do not necessarily represent those of their affiliated organizations, or those of the publisher, the editors and the reviewers. Any product that may be evaluated in this article, or claim that may be made by its manufacturer, is not guaranteed or endorsed by the publisher.

## Supplementary Material

Supplementary Table S1 Physiological characteristics of type 2 diabetes and control used in this study: Age, Body Mass Index (BMI), Fasting Blood Glucose (FBG), Fasting Insulin (FI), HbA1c, C – Peptide (CP), Cholesterol (CHL), High Density Lipoprotein (HDL), Low Density Lipoprotein (LDL), Triglycerides (TGL).

Supplementary Table S2 Step by step sequence processing information.

Supplementary Table S3 Core taxonomic composition of most dominant gut microbes at genus level with their respective relative sequence abundance.

Supplementary Table S4 Unique taxonomic composition of most dominant gut microbes at genus level with their respective relative sequence abundance for the NGT group.

Supplementary Table S5 Unique taxonomic composition of most dominant gut microbes at genus level with their respective relative sequence abundance for the T2D group.

Supplementary Figure S1 Taxonomic composition at the genus level. Relative sequence abundance of most (top 10 based) dominant gut microbes in genus level of studied samples.

Supplementary Figure S2 Dominant bacterial community between two groups (T2D and NGT). Differentially abundant OTUs within two groups are represented in Dotplot using ALDEx2. Dotplot represents class level taxonomy on the left side and genus level on the right side. The size of each dot (0, 5, 10, 15) represents centered log-ratio (clr) – transformed sequence counts.
