# Supplementary material for: Insights of Host Physiological Parameters and Gut Microbiome of Indian Type 2 Diabetic Patients Visualized via Metagenomics and Machine Learning Approaches
Source: Front Microbiol. 2022 Jul 18;13:914124. doi: 10.3389/fmicb.2022.914124 (PMC9340226; doi:10.3389/fmicb.2022.914124)
Supplement: Supplementary Table S1 — Physiological characteristics of type 2 diabetes and control used in this study: Age, Body Mass Index (BMI), Fasting Blood Glucose (FBG), Fasting Insulin (FI), HbA1c, C – Peptide (CP), Cholesterol (CHL), High Density Lipoprotein (HDL), Low Density Lipoprotein (LDL), Triglycerides (TGL). [file Table_1.DOC]

**Table S1:** Physiological characteristics of type 2 diabetes and control used in this study: Age, Body Mass Index (BMI), Fasting Blood Glucose (FBG), Fasting Insulin (FI), HbA1c, C – Peptide (CP), Cholesterol (CHL), High Density Lipoprotein (HDL), Low Density Lipoprotein (LDL), Triglycerides (TGL).

| **Sample** | **Type** | **Age** | **Sex** | **BMI (kg/m2)** | **FBG (mg/dl)** | **FI (uIU/mL)** | **HbA1c (%)** | **CP (ng/mL)** | **CHL (mg/dl)** | **HDL (mg/dl)** | **LDL (mg/dl)** | **TGL (mg/dl)** |
| --- | --- | --- | --- | --- | --- | --- | --- | --- | --- | --- | --- | --- |
| A2 | NGT | 32 | M | 22.2 | 95 | 8.26 | 6.03 | 2.53 | 199 | 48 | 133 | 208 |
| B2 | NGT | 40 | M | 28.9 | 87 | 3.02 | 6 | 1.51 | 127 | 29 | 71 | 170 |
| C2 | NGT | 23 | M | 22.7 | 70 | 18.8 | 4.6 | 2.68 | 141 | 184 | 50 | 51 |
| D2 | NGT | 43 | M | 22 | 72 | 2.89 | 5.7 | 1.03 | 197 | 33 | 153 | 146 |
| E2 | NGT | 40 | M | 20.5 | 71 | 6.98 | 5.1 | 1.71 | 139 | 35 | 95 | 98 |
| F2 | NGT | 45 | M | 38.2 | 82 | 18 | 5.8 | 5.15 | 231 | 44 | 172 | 179 |
| G2 | NGT | 35 | M | 23.6 | 89 | 2 | 5.8 | 1.25 | 197 | 38 | 137 | 150 |
| H2 | NGT | 43 | M | 24.3 | 93 | 13.4 | 5.7 | 3.11 | 276 | 44 | 185 | 164 |
| I2 | NGT | 30 | M | 27.7 | 90 | 6.27 | 5.45 | 2.52 | 241 | 31 | 123 | 651 |
| J2 | NGT | 54 | M | 23.8 | 98 | 6.06 | 5.8 | 2.35 | 351 | 32 | 239 | 252 |
| K2 | NGT | 45 | M | 38.2 | 82 | 18 | 5.8 | 5.15 | 231 | 44 | 172 | 179 |
| L2 | NGT | 44 | M | 25.8 | 69 | 8.77 | 7.6 | 3.04 | 239 | 37 | 158 | 347 |
| M2 | NGT | 45 | M | 21.88 | 93 | 9.41 | 8.8 | 3.16 | 274 | 40 | 192 | 305 |
| N2 | NGT | 52 | M | 26.7 | 70 | 6.57 | 6.8 | 3.17 | 128 | 41 | 110 | 250 |
| O2 | NGT | 45 | M | 19.1 | 100 | 3.27 | 5.2 | 1.45 | 171 | 49 | 111 | 173 |
| P2 | NGT | 42 | M | 20.9 | 110 | 3.09 | 5.5 | 1.8 | 133 | 43 | 85 | 101 |
| Q2 | NGT | 28 | M | 23.5 | 110 | 5.7 | 5.5 | 1.37 | 120 | 42 | 72 | 117 |
| A1 | T2D | 48 | M | 21.4 | 184 | 4.18 | 6.5 | 1.06 | 188 | 38 | 129 | 187 |
| B1 | T2D | 42 | M | 24.1 | 115 | 2.26 | 7.1 | 1.33 | 180 | 31 | 99 | 240 |
| C1 | T2D | 57 | M | 23.8 | 114 | 13 | 5.3 | 3.21 | 92 | 38 | 34 | 117 |
| D1 | T2D | 48 | M | 19.5 | 156 | 6.33 | 8.8 | 2.21 | 259 | 33 | 193 | 264 |
| E1 | T2D | 48 | M | 21.2 | 125 | 4.11 | 7.4 | 1.65 | 105 | 41 | 50 | 65 |
| F1 | T2D | 36 | M | 29.7 | 119 | 9.96 | 6.9 | 3 | 200 | 31 | 169 | 136 |
| G1 | T2D | 42 | M | 24 | 279 | 2.36 | 8.3 | 0.853 | 115 | 34 | 60 | 84 |
| H1 | T2D | 46 | M | 24.2 | 167 | 14.7 | 7.3 | 3.35 | 248 | 40 | 214 | 156 |
| I1 | T2D | 45 | M | 23.4 | 273 | 4.28 | 8.2 | 1.25 | 109 | 36 | 70 | 110 |
| J1 | T2D | 51 | M | 27.9 | 150 | 11.9 | 7.7 | 2.68 | 326 | 40 | 289 | 201 |
| K1 | T2D | 40 | M | 29 | 135 | 4.48 | 10.2 | 1.99 | 208 | 46 | 149 | 172 |
| L1 | T2D | 47 | M | 22.6 | 145 | 6 | 8.7 | 2.73 | 193 | 37 | 118 | 297 |
| M1 | T2D | 42 | M | 24.19 | 185 | 7.29 | 11.7 | 2.09 | 240 | 40 | 198 | 190 |
| N1 | T2D | 42 | M | 21.5 | 110 | 13.5 | 6.9 | 3.32 | 96 | 28 | 122 | 127 |
| O1 | T2D | 42 | M | 22.3 | 228.8 | 10.4 | 9.6 | 3.53 | 264 | 40 | 166 | 382 |
| P1 | T2D | 48 | M | 24.4 | 128 | 6.29 | 6.1 | 2.21 | 166 | 32 | 101 | 258 |
| Q1 | T2D | 53 | M | 24 | 248.9 | 19.1 | 10.3 | 2.92 | 203 | 49 | 139 | 142 |
