# Supplementary material for: Insights of Host Physiological Parameters and Gut Microbiome of Indian Type 2 Diabetic Patients Visualized via Metagenomics and Machine Learning Approaches
Source: Front Microbiol. 2022 Jul 18;13:914124. doi: 10.3389/fmicb.2022.914124 (PMC9340226; doi:10.3389/fmicb.2022.914124)
Supplement: Supplementary Table S2 — Step by step sequence processing information. [file Table_2.DOC]

**Table S2:** Step by step sequence processing information about total 34 samples from West Bengal.

| **Sample name** | **Raw reads** | | **Trimmed reads** | | **Merged reads** |
| --- | --- | --- | --- | --- | --- |
|  | **R1** | **R2** | **R1** | **R2** |  |
| NGT_A2 | 439815 | 439815 | 408185 | 408185 | 282099 |
| NGT_B2 | 188218 | 188218 | 177819 | 177819 | 114826 |
| NGT_C2 | 203916 | 203916 | 192060 | 192060 | 126306 |
| NGT_D2 | 359010 | 359010 | 337672 | 337672 | 215326 |
| NGT_E2 | 259562 | 259562 | 245596 | 245596 | 159454 |
| NGT_F2 | 268907 | 268907 | 255313 | 255313 | 168866 |
| NGT_G2 | 311594 | 311594 | 289635 | 289635 | 209913 |
| NGT_H2 | 122664 | 122664 | 112373 | 112373 | 80967 |
| NGT_I2 | 433617 | 433617 | 396827 | 396827 | 293562 |
| NGT_J2 | 398206 | 398206 | 370119 | 370119 | 374688 |
| NGT_K2 | 130171 | 130171 | 117736 | 117736 | 73222 |
| NGT_L2 | 92324 | 92324 | 79979 | 79979 | 57980 |
| NGT_M2 | 107082 | 107082 | 94305 | 94305 | 58283 |
| NGT_N2 | 109650 | 109650 | 97842 | 97842 | 63353 |
| NGT_O2 | 159926 | 159926 | 142419 | 142419 | 102672 |
| NGT_P2 | 109491 | 109491 | 98428 | 98428 | 72689 |
| NGT_Q2 | 108642 | 108642 | 98155 | 98155 | 72736 |
| T2D_A1 | 159569 | 159569 | 148540 | 148540 | 89266 |
| T2D_B1 | 401345 | 401345 | 378999 | 378999 | 24224 |
| T2D_C1 | 398441 | 398441 | 377084 | 377084 | 245136 |
| T2D_D1 | 411988 | 411988 | 392645 | 392645 | 269060 |
| T2D_E1 | 306343 | 306343 | 287331 | 287331 | 192160 |
| T2D_F1 | 160288 | 160288 | 152237 | 152237 | 99571 |
| T2D_G1 | 107250 | 107250 | 97719 | 97719 | 67772 |
| T2D_H1 | 117728 | 117728 | 107507 | 107507 | 76057 |
| T2D_I1 | 128132 | 128132 | 117798 | 117798 | 83202 |
| T2D_J1 | 282064 | 282064 | 260293 | 260293 | 190675 |
| T2D_K1 | 119656 | 119656 | 108908 | 108908 | 73283 |
| T2D_L1 | 120560 | 120560 | 107822 | 107822 | 79813 |
| T2D_M1 | 135500 | 135500 | 123074 | 123074 | 88987 |
| T2D_N1 | 105784 | 105784 | 93019 | 93019 | 60754 |
| T2D_O1 | 111605 | 111605 | 100007 | 100007 | 74793 |
| T2D_P1 | 151930 | 151930 | 135809 | 135809 | 93380 |
| T2D_Q1 | 109248 | 109248 | 96292 | 96292 | 65656 |
| **Total Reads** | 7130226 | | 6599547 | | 4400731 |
