# Supplementary figures and images for: Insights of Host Physiological Parameters and Gut Microbiome of Indian Type 2 Diabetic Patients Visualized via Metagenomics and Machine Learning Approaches
Source: Front Microbiol. 2022 Jul 18;13:914124. doi: 10.3389/fmicb.2022.914124 (PMC9340226; doi:10.3389/fmicb.2022.914124)

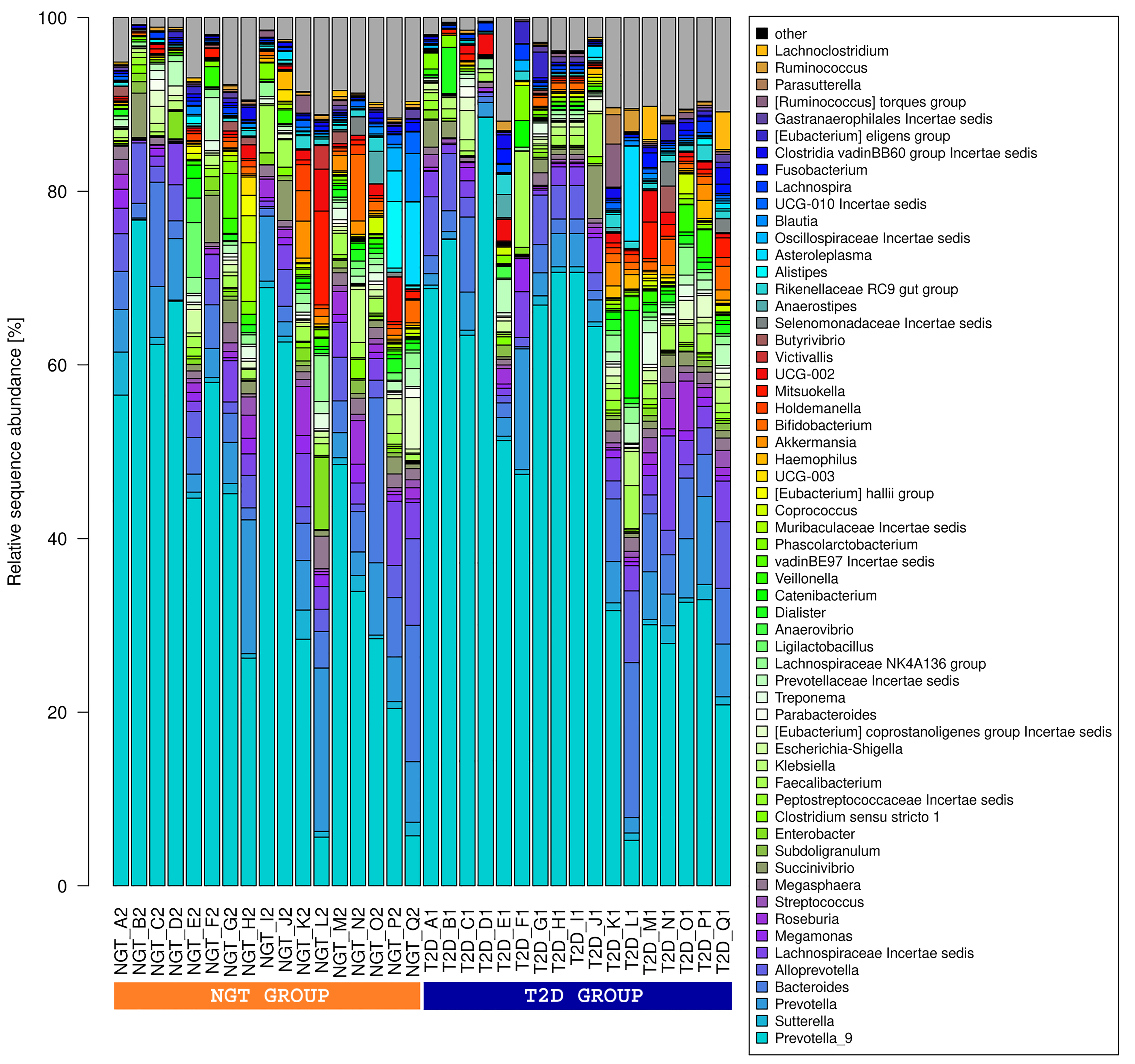

Supplement: Supplementary Figure S1 — Taxonomic composition at the genus level. Relative sequence abundance of most (top 10 based) dominant gut microbes in genus level of studied samples. [file Image_1.tiff]

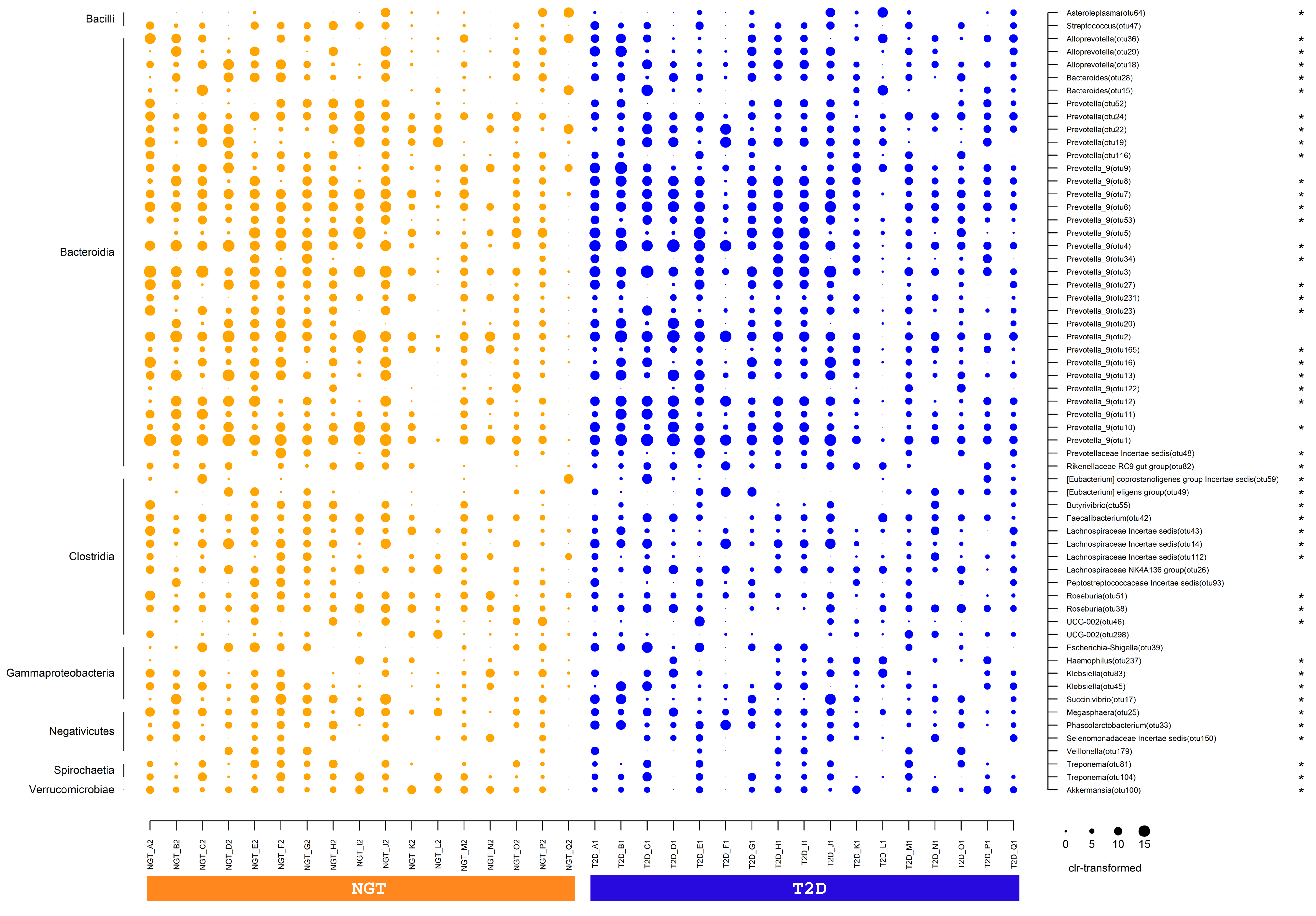

Supplement: Supplementary Figure S2 — Dominant bacterial community between two groups (T2D and NGT). Differentially abundant OTUs within two groups are represented in Dotplot using ALDEx2. Dotplot represents class level taxonomy on the left side and genus level on the right side. The size of each dot (0, 5, 10, 15) represents centered log-ratio (clr) – transformed sequence counts. [file Image_2.tiff]
